# A comparison of the efficacy and safety of complementary and alternative therapies for ulcerative colitis: A protocol for systematic review and meta-analysis

**DOI:** 10.1097/MD.0000000000021219

**Published:** 2020-07-10

**Authors:** Meiqi Lu, Ting Zhang, Zhen Lu, Wei Wang, Ting Chen, Zhiqun Cao

**Affiliations:** a The First Clinical College, Shandong University of Traditional Chinese Medicine; b Department of Pharmacy, Shandong Rehabilitation Hospital; c The Affiliated Hospital of Shandong University of Traditional Chinese Medicine, Jinan, Shandong Province, China.

**Keywords:** complementary and alternative therapies, meta-analysis, protocol, systematic review, ulcerative colitis

## Abstract

**Background:**

The incidence of ulcerative colitis (UC) is increasing year by year worldwide, and it is listed as one of the refractory diseases by World Health Organization. In addition to typical intestinal manifestations such as abdominal pain, diarrhea, mucus, pus, and bloody stool, it can also accompany multiorgan and multisystem extraintestinal manifestations, seriously affecting the life and work of patients. Furthermore, UC patients with a tremendous psychological pressure and affects their physical and mental health. In recent years, many complementary and alternative therapies have been used for treatment of UC, but only pair-wised drugs have been evaluated in the traditional meta-analyses and some results are inconsistent. Consequently, it is essential to propose a protocol for systematic review and meta-analysis to discuss the efficacy and safety of complementary and alternative therapies in the treatment of UC.

**Methods:**

We will search Chinese and English databases comprehensively and systematically from the establishment of databases to May 2020, free of language or publication restrictions. All randomized controlled trials on complementary and alternative therapies for UC will be included. Two researchers will independently screen titles, abstracts, full texts, and extract data, then assess the bias risk of each study. We will conduct pairwise meta-analyses and Bayesian network meta-analyses to the relative outcomes of the efficacy and safety. Data analysis will use STATA and WinBUGs 1.4.3 software in this meta-analysis.

**Results:**

This study will evaluate the efficacy and safety of complementary and alternative therapies for UC based on changes in symptoms, clinical efficacy, quality of life and adverse events.

**Conclusion:**

This study will provide evidence for whether complementary and alternative therapies are beneficial to the treatment of UC. In order to provide reliable evidence-based medicine for clinical practice.

**INPLASY registration number:**

INPLASY202060015

## 1 Introduction

Ulcerative colitis (UC) is a chronic nonspecific inflammatory disease involving colon and rectum,^[1]^ which is one of the inflammatory bowel diseases. The clinical manifestations of UC mainly include abdominal pain, diarrhea, bloody purulent stool, hematochezia, fever, joint pain, and so on. Worldwide, joint involvement is the most common extraintestinal manifestation of ulcerative colitis^[2]^; in addition, it can also be accompanied by extraintestinal manifestations such as skin mucosa, eyes, hepatobiliary, and bone.^[3–6]^ UC can occur at any age.^[7]^ There is no significant sex difference between men and women.^[8]^ The prevalence of UC in urban population is relatively high.^[9]^ So far, the pathogenesis of UC is not very clear, but it has been confirmed that genes, environment, intestinal microorganisms, and autoimmune factors are involved in the pathogenesis process of UC.^[1,10,11]^ A prospective study^[12]^ found that dietary factors are closely related to the onset of UC, and meat in the diet (especially red meat and processed meat, alcoholic beverages, and protein intake) is also associated with an increased likelihood of recurrence in UC patients. Occurrence of this disease has a serious impact on the normal life, work, and mental health^[13]^ of patients. Once the disease occurs, the course of disease is very long and difficult to cure. Patients with long course of UC have higher risk of cancer.^[14]^ Because of its high incidence and mortality of colon cancer,^[15–17]^ it is very important to give effective treatment. According to statistics, the highest prevalence of UC in Europe is 505 per 100,000 people and 249 per 100,000 people in North America.^[18]^ In Asia and Japan, the prevalence of UC is 172.9 per 100000 people, and in the past 2 decades, the prevalence of UC has increased year by year.^[19]^ The conventional methods of the treatment for UC patients mainly include aminosalicylic acid, glucocorticoid, immunosuppressive agents, biological agents, and operation, but the long-term application has more side effects. The advantages of complementary and alternative therapy are safety, low toxicity, effectiveness, and economic benefits. Chinese herbal medicine, as a kind of complementary and alternative therapy, has been proved to be effective in the treatment of UC.^[20,21]^ Acupuncture, moxibustion, massage included in complementary, and alternative therapy are all common external treatment methods, which are carried out under the guidance of traditional Chinese medicine theory. They have a long history of disease prevention and treatment. For the long course and refractory of UC, it has been a main task of clinicians and researchers to cure it in the worldwide for a long time.

This disease was once called “dysenteric diarrhea” and “dysentery” in ancient Chinese classics. Modern Chinese medicine theories mostly attribute it to the categories of “intestinal wind,” “intestinal mass,” “protracted dysentery,” and “stagnation.”^[22]^ Traditional Chinese medicine believes that the occurrence of this disease is mainly due to external pathogenic factors, diet injuries, emotional disorders, congenital weakness of the spleen, and stomach,^[23]^ which leads to damp heat, phlegm turbidity, blood stasis, evil toxin blocking in the intestinal tract, resulting in abdominal pain, diarrhea, bloody stool, and other clinical manifestations. Complementary and alternative therapies include traditional Chinese medicine, acupuncture, moxibustion, massage, and so on. Now many clinical trials have confirmed that complementary and alternative therapies are effective in the treatment of UC, but the quality of these trials is uneven, and they do not have a high persuasiveness, which will affect the scientificity of the research conclusions to a certain extent, and it is difficult to be generally recognized by the medical community. The purpose of this study is to provide convincing and comprehensive evidence for comparing the efficacy and safety of complementary and alternative therapies in the treatment of UC.

## 2 Methods and analysis

### 2.1 Study registration

This protocol has been registered in the International Platform of Registered Systematic Review and Meta-analysis Protocols (INPLASY.COM) and the registration number is INPLASY202060015. This study has followed the guidelines of Preferred Reporting Items for Systematic Review and Meta-Analysis Protocols (PRISMA-P).^[24]^

### 2.2 Inclusion criteria

#### 2.2.1 Type of research

This study includes all relevant randomized controlled trials (RCTs) of complementary and alternative therapies for UC published in Chinese or English, regardless of allocation hidden or blinded.

#### 2.2.2 Types of patients

The population includes patients with UC diagnosed according to any recognized diagnostic criteria which are internationally or nationally authorized (such as ACG Clinical Guideline: Ulcerative Colitis in Adults,^[25]^ Consensus of Experts in Diagnosis and Treatment of Ulcerative Colitis^[26]^). We do not impose restrictions on sex, race, region, or other characteristics.

#### 2.2.3 Interventions

The complementary and alternative therapies for treating UC include acupuncture, moxibustion, massage, Chinese herbal medicines and topical warm treatment, regardless of disposal method, and duration. Whether it is used in combination with other treatments or used alone. The control group does not use complementary and alternative therapies or drug therapy, and the treatment was commonly used clinical drugs such as aminosalicylic acid preparation.

#### 2.2.4 Outcomes

The primary outcome measures are improvements in clinical symptoms such as abdominal pain, diarrhea, bloody purulent stool and clinical effectiveness, with secondary outcomes being quality of life, and the incidence of adverse events.

### 2.3 Search strategy

We will use the computer to search the following electronic bibliographic databases: PubMed, the Cochrane Controlled Trials Central Register System (CENTRAL) Cochrane Library, EMBASE, China National Knowledge Infrastructure (CNKI), Wanfang Database, VIP, and Chinese Biomedical Literature database (CBM). The retrieval time is from the establishment of databases to May 2020. We will apply a combination of MeSH terms and free-text to search and adjust the search strategy according to the characteristics of each database. PubMed's detailed search strategy is shown in Table 1.

**Table 1:**
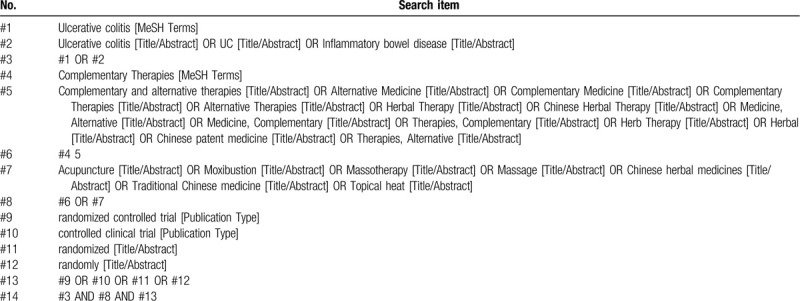
Details of the search strategy of PubMed.

### 2.4 Study selection and data extraction

The 2 reviewers will independently select the literature and extract the data according to the established retrieval strategy, and discuss or consult the third reviewer to make a decision in case of disagreement. During the selection and identification of studies, the 2 reviewers first read the title and abstract of each literature, excluding unrelated studies. The second step is to read the full text of the literature initially identified for inclusion. During the data extraction, the Microsoft Excel data extraction form will be used to extract the data from the literature included. We will attempt to extract the following data information from each study: first author, title, country/region, year of publication, study design, sample size, intervention approach, outcome indicators, adverse events. If the required data are missing, we will try to contact the literature authors to ensure the accuracy of the relevant information.

### 2.5 Risk of bias assessment

Risk of bias in the included studies will be assessed by the Cochrane Risk of Bias Tool^[27]^ according to the Cochrane Handbook 5.1.0 for Systematic Reviews of Interventions, which consists of 7 items of bias relevant to the quality of RCTs. The criteria to be assessed include the following domains: random sequence generation (selection bias), allocation concealment (selection bias), blinding of participants and personnel (performance bias), blinding of outcome assessment (detection bias), incomplete outcome data (attrition bias), selective reporting (reporting bias), and other bias. An assessment of risk of bias will be made for the included studies based on the following thre3 levels: “low risk of bias,” “unclear risk of bias,” “high risk of bias.” Such an evaluation process will be independently performed by 2 researchers, and when differences arise, a third person will be required to participate in the discussion to determine the risk of bias.

### 2.6 Statistical analysis

#### 2.6.1 Pairwise meta-analyses

Using Revman 5.3 software provided by Cochrane Collaboration Network, meta-analyses were carried out for the included researches. When *P* > .05, we generally determined that there is no heterogeneity among the studies, fixed-effects model is selected for analysis, otherwise random-effects model is selected. In addition, we also use *I*^2^ for quantitative analysis of heterogeneity^[28]^; it is generally considered that *I*^2^ > 50% indicates the existence of substantial heterogeneity. When there is homogeneity among the studies, the fixed-effects model analysis is used; when there is heterogeneity between the studies, the random-effects model analysis is used; the statistics of efficacy index analysis use odds ratio (OR), continuous variables use weighted mean difference, and 95% confidence interval is given. Potential publication bias will be analyzed by inverted funnel plots.

#### 2.6.2 Network meta-analyses

Network meta-analyses can be used for existing direct comparisons of evidence for summary, but also lack of evidence in head-to-head comparisons. At the same time, indirect comparisons provide useful information and can also be used for various interventions. The results are sorted to effectively answer the decision-making problem of the intervention effect. We will execute Bayesian network meta-analysis of Markov chain Monte Carlo methods^[29]^ in WinBUGS 1.4.3. The binary data are expressed by the OR and its 95% prediction interval. Given that it could not generate graphics, the mvmeta command will be used in Stata 14.0 for graphics related analysis.^[30]^ The direct comparison and indirect comparison between different interventions were presented by drawing a network diagram. *χ*^2^ test was used to analyze the overall heterogeneity of 2-arm research and network. If the total network *I*^2^ ≤50%, then the heterogeneity is small, fixed-effects model can be selected for network meta-analysis; if the total network *I*^2^ > 50%, then the heterogeneity is large, analyze the causes of heterogeneity, and then select random-effect model for network meta-analysis after excluding the heterogeneity factors. In Stata 14.0 software, the results of intervention measures were ranked, and the surface under the cumulative ranking curves (SUCRA)^[31]^ and mean ranks (MRs) were used as evaluation indexes. The SUCRA value is a probable indicator of the pros and cons of the intervention. The closer the value is to 100%, the greater the likelihood of becoming optimal. The closer the MR is to 0, the better the efficacy of the intervention. Publication bias analysis was performed using an inverted funnel plot. The difference was statistically significant (*P* < .05).

#### 2.6.3 Measures for inconsistency

When there are 3 treatments that make up a loop, we will evaluate the inconsistencies between them. For the same comparison, we will estimate the consistency between direct and indirect effects. Using the mvmeta command in STATA, the node splitting method will be used to evaluate the consistency of the whole network.

#### 2.6.4 Subgroup analysis

If the included evidence is rich, we will conduct a subgroup analysis of the study, such as: the severity of the disease and the different treatments.

#### 2.6.5 Sensitivity analysis

This process will be carried out by eliminating each low-quality study. After the study was excluded, the combined effect was reestimated and compared with the results of meta-analysis before exclusion. If the heterogeneity does not change after excluding each literature, we think our conclusion is stable; otherwise, if the heterogeneity changes, the excluded literature may be the source of heterogeneity.

#### 2.6.6 Assessment of evidence quality

The evidence quality of the study will be assessed by the Grading of Recommendations Assessment, Development and Evaluation (GRADE) framework.^[32]^ The 4 levels of the GRADE for the quality of evidence are very low quality, low quality, moderate quality, and high quality.

## 3 Discussion

UC is a chronic intestinal inflammatory disease. The disease is mainly centered on the occurrence of chronic, nonspecific inflammatory lesions in the large intestinal mucosa and submucosa. Mucosal epithelium and glands are destroyed, proliferated, or atrophied, lymphocytes, neutrophils and monocytes infiltrate, and crypt abscesses form. Its main pathological manifestations are mucosal hyperemia, erosion, ulcers, and proliferative changes.^[8,25,33]^ The pathogenesis of this disease is complex, no new progress has been made in treatment methods, and the treatment effect is not unsatisfactory. It is listed as one of modern refractory diseases by the World Health Organization.^[34]^

Complementary and alternative therapies, as a new treatment with broad development prospects, have obvious effects in the treatment of UC. The traditional meta-analyses on complementary and alternative therapies only compare the 2 treatments or 2 drugs, and the results of some studies are contradictory. Therefore, this systematic review compares the efficacy and safety of complementary and alternative therapies in the treatment of UC. And we hope our study will provide guidance for better options in the clinical treatment of alleviating the patient's discomfort, improving the quality of life, and so on. For this purpose, multicenter, multisample, high-quality clinical research and data are still necessary to evaluate the efficacy and safety of complementary and alternative therapies for UC.

### 3.1 Uncited Reference

^[33]^.

## Author contributions

**Conceptualization:** Meiqi Lu, Zhiqun Cao.

**Data curation:** Meiqi Lu, Ting Zhang, Wei Wang.

**Formal analysis:** Meiqi Lu, Zhen Lu.

**Funding acquisition:** Zhiqun Cao, Wei Wang.

**Methodology:** Meiqi Lu, Ting Zhang, Zhen Lu.

**Resources:** Ting Chen.

**Software:** Meiqi Lu, Ting Chen, Ting Zhang.

**Supervision:** Wei Wang.

**Writing – original draft:** Meiqi Lu.

**Writing – review & editing:** Meiqi Lu, Zhiqun Cao.
